# Outcome of Neoadjuvant Chemotherapy on Local Recurrence and Distant Metastasis of Oral Squamous Cell Carcinoma: A Retrospective Study

**Published:** 2016-09

**Authors:** Reza Tabrizi, Ata Garajei, Ehsan Shafie, Samira Jamshidi

**Affiliations:** 1 Dept. of Oral and Maxillofacial Surgery, Shahid Beheshti University of Medical Sciences, Tehran, Iran.; 2 Dept. of Oral and Maxillofacial Surgery and Research Director, Craniomaxillofacial Research Center, School of Dentistry, Tehran University of Medical Sciences, Tehran, Iran.; 3 Postgraduate Student, Dept. of Oral and Maxillofacial Surgery, School of Dentistry, Shiraz University of Medical Sciences, Shiraz, Iran.; 4 Postgraduate Student, Dept. of Oral and Maxillofacial Medicine, School of Dentistry, Shiraz University of Medical Sciences, Shiraz, Iran.

**Keywords:** Squamous Cell Carcinoma Oral, Metastasis, Recurrence, Chemotherapy

## Abstract

**Statement of the Problem:**

Neoadjuvant chemotherapy (NCH) is controversial in the treatment of oral squamous cell carcinoma (OSCC).

**Purpose:**

The aim of this study was to evaluate the efficacy of NCH on OSCC prognosis.

**Materials and Method:**

In this retrospective cohort study, 94 patients were studied in two groups. The patients in group 1 received NCH before the surgery, and those in group 2 underwent resection without any chemotherapy prior to surgery. The employed NCH agents consisted of cisplatin in combination with 5-fluorouracil in two treatment courses. Tumor size, lymph node involvement, age, and follow-up time were considered as variable factors of the study. Local recurrence (LR) and distant metastasis (DM) were outcomes of the study.

**Results:**

Comparison of LR and DM in various tumor sizes demonstrated no significant difference between the two groups (*p*> 0.05). Analysis of the data did not show any statistically significant difference between the groups for LR in subjects with N0, N1 and N2. Each one-year increase in age was associated with 10% increase in the hazard ratio (HR) (HR distance metastasis Y/N = 1.10, *p*= 0.05). In the same analysis, when considering LR as a dependent factor, LR risk in N2 was 3 times more than in N1 (*p*= 0.02). LR risk in N3 was 5 times more than in N1 [HR local recurrence (*p*= 0.006).

**Conclusion:**

Based on our results, neoadjuvant chemotherapy with combination of cisplatin and 5-fluorouracil may not improve prognosis of OSCC. However, further studies are suggested to assess other neoadjuvant chemotherapy protocols in OSCC patients.

## Introduction

Oral squamous cell carcinoma (OSCC) is the most frequent malignant tumor of the oral cavity around the world.[1] Conclusive local therapies defined as surgery, radiation therapy, or both are the conventional treatment approaches in OSCC. However, OSCC is associated with high rates of local and distant recurrences. Treatments can cause considerable morbidity, including loss of swallowing ability, and also tongue and larynx dysfunction. Improved cure rates and functional outcomes have led to adding chemotherapy to the treatment of OSCC. Combined modality of the treatment has advantages such as functional organ preservation in patients with locoregionally advanced head and neck cancer.[2] Neoadjuvant chemotherapy in OSCC has been investigated. Despite the absence of absolute scientific evidence; it is often used in clinical practice based on biological and practical considerations.[3]

It has been shown that untreated OSCC is chemo-sensitive; besides, multiple agent chemotherapy has a response rate in approximately 70-80% of tumors.[3] Some studies showed that induction chemotherapy decreased distant metastasis (DM) without improvement of survival rates.[3] Other studies demonstrated a benefit of neoadjuvant chemotherapy in OSCC.[4-5] The effect of neoadjuvant chemotherapy on local recurrence (LR) rate is still controversial. Few studies focused on outcomes of neoadjuvant chemotherapy in treatment of OSCC in various tumor sizes and lymph node involvements.[6]

The aim of this study was to evaluate the effect of neoadjuvant chemotherapy on decrease of LR and DM in OSCC. 

## Materials and Method

The samples in this retrospective cohort study were derived from the population of patients who referred to the hospitals of Tehran University of Medical Sciences and Shiraz University of Medical Sciences between September 1, 2004 and September 31, 2013. Subjects were eligible if they had biopsy-proven OSCC, had undergone tumor resection with post-surgery radiation therapy, and participated in the study’s follow-ups. Patients were excluded from the study if they had a metastatic lesion initially, extra oral involvement, or pre-operation radiotherapy. Those with OSCC in floor of the mouth, tongue, or the mandible were studied.

Patients were studied in two groups. The patients in group 1 had been received neoadjuvant chemotherapy before the surgery and those in group 2 have been undergone surgical resection without any chemotherapy before their surgery.

The surgical approaches consisted of tumor resection with 1 cm safety margin and also marginal resection or hemimandibelectomy. Uni- or bilateral selective neck dissection was performed in all subjects. 

All patients have been received radiotherapy for four to eight weeks after the surgery and doses between 60 and 70 Gy have been applied (180-200 cGy/d, 5 days/ week). 

The neoadjuvant therapy agents consisted of cisplatin (20 mg/m^2^/d continuous infusion) in combination with 5-fluorouracil (800 mg/m^2^/d CI) in two treatment courses.

The LR and DM were documented in each patient and have been proven by histopathological evaluation. Tumor size and lymph node have been documented according to the tumor staging system for SCC (Table 1).[7]

**Table 1:** SCC staging system

N0: No Discernible nodes. N1: Ipsilateral nodes, <3 cm in diameter. N2: Single ipsilateral node, 3-6 cm in diameter, Multiple ipsilateral nodes, <6 cm in diameter, contralateral or bilateral nodes, <6 cm in diameter. N3: Node or Nodes> 6cm in diameter
M0: Initially without distant metastasis. M1: Initially with distant metastasis.
Stage I: T1N0M0 Stage II: T2N0M0 Stage III: T3N0M0, T1, T2 or T3 N1M0 Stage IV: T4 N0 or N1, M0 Any T1N2 or N3, M0 Any T, any N, M1

Tumor size, lymph node involvement, age and follow-up time were considered as variable factors of the study. The LR and DM were outcomes of the study.

A vertical incision was made to split the lower lip at the midline and was extended posteriorly 4 cm inferior to the lower border of the mandible. The lesion was resected with a 1-cm safety margin. Frozen section was done for confirmation of sufficient safety margin during the surgeries. A selective neck dissection was performed in the zone I, II and III of the neck.

The statistical analyses were performed by using SPSS statistical package, version 19 (IBM; USA). Chi-square test was used to compare the DM and LR between the two groups in various tumor sizes and lymph node involvements. Cox regression test was applied to investigate the time effect of variable factors on DM and LR (significance level ≤0.05). 

## Results

Ninety-four patients (63 males and 31 females) were studied in two groups. Group 1 consisted of 30 males and 17 females and group 2, 33 males and 14 females. There was no significant difference between the two groups in terms of gender (*p*= 0.66). Evaluation of data showed that LR was more frequent in females than males (*p*= 0.027). There was no difference between males and females in DM (*p*= 0.57). The mean age was 63.70±11.18 years in group 1 and 63.34±10.61 years in group 2. The mean follow-up time was 32.15±19.24 months in group 1 and 32.74±19.09 months in group 2. Comparison of age and follow-up time between the two groups did not show any statistically significant differences (*p*> 0.05). (Table 2)

**Table 2:** Comparison of variable factors between the two groups

Gender	M(30), F(17)	M(33), F(14)	*p*˃ 0.05*
Age (years)	63.70±11.18	63.34± 10.61	*p*˃ 0.05**
Follow up (months)	32.15±19.24	32.74 ± 19.09	*p*˃ 0.05**

*Chi-square test **Independent t-test

Comparison of LR in various tumor sizes demonstrated no statistically significant difference between the two groups (*p*> 0.05). (Table 3)

**Table 3:** Comparison of LR in various tumor sizes between the two groups

T1	LR(0) ,WLR(2)	LR(1), WLR(3)	*p*= 0.67
T2	LR(2) ,WLR(8)	LR(1),WLR(8)	*p*= 0.54
T3	LR(9),WLR(10)	LR(8).WLR(9)	*p*= 0.56
T4	LR(15),WLR(1)	LR(11).WLR(5)	*p*= 0.086

Analysis of data did not show any statistically significant differences between the groups concerning LR in subjects with N0, N1 and N2. (Table 4)

**Table 4:** Comparison of LR in various lymph node involvements between the two groups

N1	LR(4),WLR(16)	LR(2),WLR(15)	*p*=0.413
N2	LR(5),WLR(15)	LR(10),WLR(14)	*p*= 0.21
N3	LR(7),WLR(0)	LR(5),WLR(1)	*p*= 0.46

**Figure 1:**
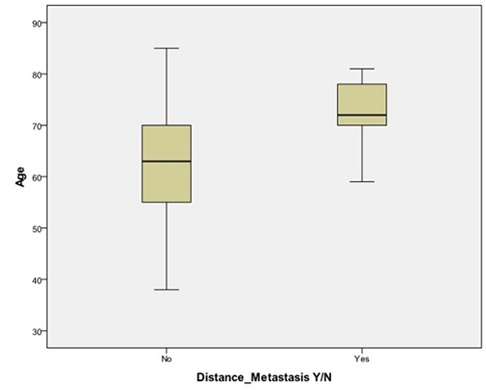
The effect of age on DM

**Figure 2:**
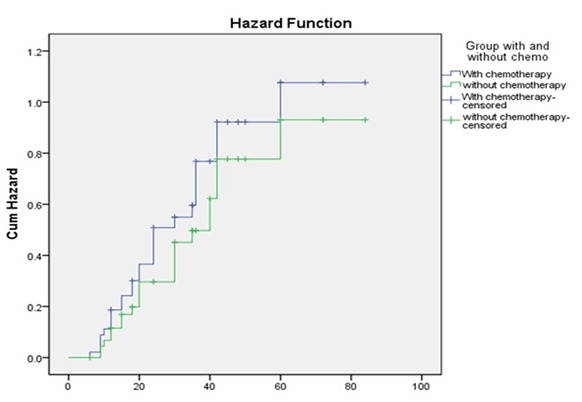
Comparison of LR risk between the two groups (Kaplan- Meier plot)

Considering DM as a dependent factor, age, gender, groups, tumor size, and node involvement as independent factors, cox regression revealed that age had a correlation with DM. Each one-year increase in age increased the hazard ratio (HR) by 10% (HR of distant metastasis Y/N = 1.10, *p*= 0.05). (Figure 1) In the same analysis, considering LR as a dependent factor, LR risk in N2 was 3 times more than that in N1 [)HR Local recurrence (N2 vs. N1) = 3.1, *p*= 0.02]. LR risk in N3 was 5 times more than that in N1 [HR Local recurrence (N3 vs. N1) = 5.2, *p*=0.006, Figure 2].

**Figure 3:**
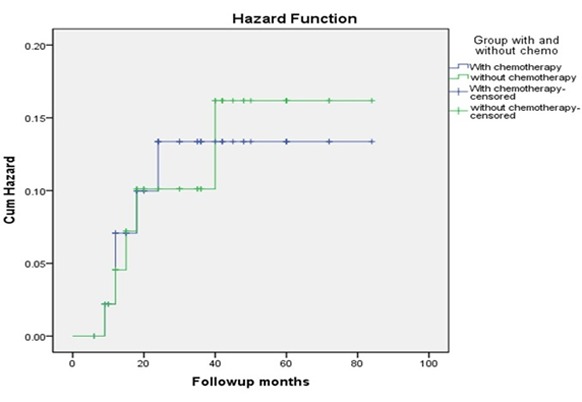
Comparison of DM risk between the two groups (Kaplan- Meier plot)

Five subjects had DM in each group. The results did not demonstrate any difference for DM between the two groups during follow-up time (*p*=0976). (Figure 3)

LR and DM were the same in follow-up period in the two groups according to the Log-Rank (Mantel-Cox) test and Kaplan-Meier curves. 

## Discussion

Treatment of advanced OSCC classically includes surgical resection with postoperative adjuvant radiotherapy. Despite this aggressive dual modality therapy, the disease outcome has remained constant at 30% local or regional disease recurrence, 25% DM, and 40% five-year survival.[8] Neoadjuvant chemotherapy has been studied over the past two decades and used for patients with locally advanced squamous cell carcinoma of the head and neck.[9] Concurrent radiochemotherapy remains the standard treatment for patients with unresectable, nonmetastatic locoregionally advanced SCC of the head and neck.[10] However, the use of neoadjuvant therapy in resectable or low stages of OSCC is controversial.[11]

The study volume (94 cases) was reasonable compared to the previous studies in which Maggiore *et al*.[12] examined 89 patients and Park *et al*.[13] studied 114 patients. Tumor size, lymph node involvement, age and follow-up time were the variables in this study. Zhang *et al.*[14] considered the demographic, pathologic, treatment, and survival data in their study. Chinn *et al*.[15] studied 19 patients with resectable stages III and IV of OSCC. They determined age, gender, pretreatment stage, T and N classifications, smoking status, alcohol consumption, or tumor subtype on the basis of American Joint Committee on Cancer. They detected that primary surgical treatment showed significantly better survival and locoregional control.[15]

It has been shown that neoadjuvant chemoradiotherapy with 40 Gy and concurrent low-dose cisplatin monotherapy followed by selective surgery is an appropriate and reliable therapy concept; which results in encouraging overall and disease-free survival rates in responders. Non-responders might profit from intensified systemic therapy approaches.[16] The majority of phase III clinical trials of neoadjuvant chemotherapy focus on unresectable or locally advanced OSCC.[17-18] In our study, neoadjuvant chemotherapy did not improve LR and DM in various tumor sizes and N0 and N1. Furthermore, minimal improvements (not statistically significant) in T4 tumor sizes for LR were noticed in subjects who received neoadjuvant chemotherapy.

Licitra *et al*.[19] demonstrated that adding primary chemotherapy to standard surgery did not improve survival. However, in his study, primary chemotherapy seemed to play a role in reducing the number of patients who needed to undergo mandibulectomy and/or radiation therapy. Variations in the criteria used to select the patients for these treatment options make it difficult to generalize these results. But, it appeared that using preoperative chemotherapy to spare destructive surgery or radiation therapy in patients with advanced, resectable oral cavity cancer was reasonable. It is well known that primary surgery with adjuvant radiation or chemo-radiation was complementary to primary chemoradiotherapy for nonresectable tumors. Ongoing studies are sorting out the role of induction chemotherapy in the current context of clarifying optimal multimodal treatment.[20]

In one study, 276 consecutive patients with OSCC stages III and IV (T2: 13.0%; T3: 16.7%; T4: 70.3%; N0: 29.7%; N1: 20.3%; N2: 45.3%; N3: 4.7%; stage III: 16.3%; stage IV: 83.7%) received preoperative radio-chemotherapy (50Gy, mitomycin and 5-fluorouracil) and radical locoregional resection. The median surveillance period was 101.4 months (24-202 months). The 5-year overall survival probability was 53.9%. The 5-year local control probability was 70.2%. The authors concluded that preoperative treatment of patients with oral and oropharyngeal cancer was a reliable therapy.[21] The main limitation of that study was the study design (a retrospective cohort study) and the authors did not compare the survival rate with that of patients who did not receive radiochemotherapy.

Another retrospective cohort study was performed on 222 patients who underwent multimodal therapy between 1990 and 2000.[22] The eligible patients had oral and oropharyngeal SCC stages II-IV (T2: 33.3%; T3: 12.6%; T4: 54.1%; N0: 45.9%; N1: 17.6%; N2: 33.3%; N3: 3.2%; stage II: 21.1%; stage III: 14.9%; stage IV: 64%). The patients received preoperative radio-chemotherapy consisting of mitomycin C (15-20 mg/m^2^, day 1) plus 5-fluorouracil (750 mg/m^2^/24 h-infusion, days 1-5) and concomitant radiotherapy for a total dose of 50 Gy. Radical locoregional en bloc-resection according to the pretherapeutic tumor extension was carried out in all patients. After a median surveillance period of 72.3 months (24-152 months), 131 patients (59%) were alive and 91 patients (41%) expired. Twelve patients (5%) died postoperatively, 46 patients (21%) died due to tumor recurrence, and 33 (15%) deaths were not directly related to the primary tumor. Overall, the survival probability was 76% after two years and 62% after five years. Two- and five-year local control probabilities were 88 and 81%, respectively. The authors concluded that the multimodal concept was an effective therapy offering excellent survival and local control probability.[22]

Recently, it is claimed that current data would not support the use of induction chemotherapy before planned surgical intervention for advanced oral cavity and oropharyngeal tumors. Currently, for patients with locoregionally advanced unresectable disease, concomitant chemo-radiation is considered as the standard of care in waiting for results of the few ongoing studies that hopefully will clarify the role of induction of 5-fluorouracil TPF before either concomitant chemo-radiation or bio-radiation.[18] According to the studies over the past decade, it is unclear whether neoadjuvant chemotherapy is beneficial in treatment of OSCC.[23-24] Eric *et al*.[25] studied the effect of neoadjuvant chemoradiotherapy and radical resection on advanced OSCC. They used radio-chemotherapy consisting of 39.6 Gy in daily fractions of 1.8 Gy and concomitant carboplatin (70 mg/m^2^ days 1-5). They concluded that neoadjuvant chemoradiotherapy with subsequent radical surgery can be recommended as an effective and safe treatment for primary resectable advanced tumors of the oral cavity.[6]

Two agents were used in our study for chemotherapy. It was a main limitation of this study. Use of a combination of other chemotherapy agents may change the outcome of the study. Moreover, increasing the number of cases enhances the reliability of the study. Further clinical trials are necessary to evaluate various chemotherapy protocols in treatment of OSCC patients. 

## Conclusion

Based on our results, neoadjuvant chemotherapy with combination of cisplatin and 5-fluorouracil may not improve the prognosis of OSCC. However, further studies are suggested to assess other neoadjuvant chemotherapy protocols in OSCC patients. 
